# Dual Memory LSTM with Dual Attention Neural Network for Spatiotemporal Prediction

**DOI:** 10.3390/s21124248

**Published:** 2021-06-21

**Authors:** Teng Li, Yepeng Guan

**Affiliations:** 1 School of Communication and Information Engineering, Shanghai University, Shanghai 200444, China; 15668188030@163.com; 2 Key Laboratory of Advanced Display and System Application, Ministry of Education, Shanghai 200072, China

**Keywords:** spatiotemporal prediction, dual memory LSTM, dual attention, historical representations

## Abstract

Spatiotemporal prediction is challenging due to extracting representations being inefficient and the lack of rich contextual dependences. A novel approach is proposed for spatiotemporal prediction using a dual memory LSTM with dual attention neural network (DMANet). A new dual memory LSTM (DMLSTM) unit is proposed to extract the representations by leveraging differencing operations between the consecutive images and adopting dual memory transition mechanism. To make full use of historical representations, a dual attention mechanism is designed to capture long-term spatiotemporal dependences by computing the correlations between the current hidden representations and the historical hidden representations from temporal and spatial dimensions, respectively. Then, the dual attention is embedded into DMLSTM unit to construct a DMANet, which enables the model with greater modeling power for short-term dynamics and long-term contextual representations. An apparent resistivity map (AR Map) dataset is proposed in this paper. The B-spline interpolation method is utilized to enhance AR Map dataset and makes apparent resistivity trend curve continuous derivative in the time dimension. The experimental results demonstrate that the developed method has excellent prediction performance by comparisons with some state-of-the-art methods.

## 1. Introduction

Spatiotemporal prediction is learning representations in an unsupervised manner from unlabeled video data and using them to execute a prediction task, which is a typical computer vision task. Currently, the spatiotemporal prediction has been applied to some tasks successfully, such as future prediction of object locations [1,2], anomaly detection [3], and autonomous driving [4]. Deep learning-based models take a leap over the traditional approaches because they have learned adequate representations from high-dimensional data. Deep learning methods fit perfectly into the spatiotemporal prediction task, which could extract spatiotemporal correlations from video data in a self-supervised fashion. However, spatiotemporal prediction is still a challenging task due to the problem of extracting representations inefficiently and the lack of long-term dependencies. For example, Convolutional LSTM (ConvLSTM) [5] has been developed to further extract temporal representations but it ignores spatial representations. Some methods [6,7] have achieved accurate prediction results, but they cause representation loss. The method of adversarial has been applied in prediction tasks [8,9]. However, they [8,9] are significantly dependent on the unstable training process.

A novel dual memory LSTM with dual attention neural network (DMANet) has been proposed for spatiotemporal prediction in this paper to solve the mentioned problems. A dual memory LSTM (DMLSTM) unit based on ConvLSTM [5] has been developed for DMANet to perform spatiotemporal prediction. It can be applied to get representations of motion by differencing adjacent hidden states or raw images appropriately. Besides it has dual memory structures to store spatial information and temporal information. A dual attention mechanism is proposed and embedded into the DMLSTM unit to extract long-term feature dependencies from temporal and spatial dimension, respectively, which enables the developed model to capture longer complex video dynamics. Compared with the above spatiotemporal prediction methods, the main contributions of this paper are as follows. Firstly, a novel DMLSTM unit has been proposed to perform extract representations, which can be applied for spatiotemporal prediction by leveraging differencing operations between the consecutive images and adopting dual memory transition mechanism. Secondly, a dual attention mechanism is developed to get the long-term frame interactions. The long-term frame interactions are captured by computing the correlation between the currently hidden representations and the historical hidden representations from the temporal and spatial dimension, respectively. Finally, an important contribution is that the DMANet combines both the advantages. Such architectural design enables the model with greater modeling power for short-term dynamics and long-term contextual representations. The proposed method is evaluated at some challenging datasets with different methods. It achieves excellent performance by comparison with some state-of-the-art methods. The experimental results show that the proposed method has excellent spatiotemporal prediction performance.

The rest of this article is organized as follows. Related work is discussed in Section 2. The dual memory LSTM with dual attention mechanism is described in Section 3. Experimental results and analyses are discussed in Section 4 and followed by conclusions in Section 5.

## 2. Literature Review

Over the past decade, many methods have been proposed for spatiotemporal prediction. Recurrent neural network (RNN) [10] with the long short-term memory (LSTM) [11] has been increasingly applied to prediction task due to its capabilities for learning representations of video sequence. In recent years, the LSTM framework based on a sequence-to-sequence model [12] has been adapted to video prediction. Still, the accuracy of prediction is limited due to the fact that these framework methods [12] only capture temporal variations. In order to further extract video representations, ConvLSTM [5] replaces fully connected operations with convolution operations in recurrent state transitions. A deep-learning-based framework [13] is proposed to reconstruct the missing data to facilitate analysis with spatiotemporal series. However, it will increase the extra computational cost and lower the prediction efficiency. The bijective gated recurrent unit is introduced in [14], which exploits recurrent auto-encoders to predict the next frame in some cases. A multi-output and multi-index of supervised learning [15] method with LSTM [11] is proposed for spatiotemporal prediction, which can model the long-term dynamics. In pursuit of alleviating gradient vanishing, convolutional LSTM extended by [6,7] introduces a zigzag memory flow and gradient highway unit (GHU). An updated deep learning-based method has been used for improving prediction capability. A version of ASAP called the “ASAP deep system”, is proposed in [16]. Optical flow warping and RGB pixel synthesizing algorithms [17] has been exploited to perform spatiotemporal prediction. Memory-in-memory network (MIM) is proposed for prediction task in [18]. Its difference from the above-mentioned recurrent models is that MIM [18] applies differencing in memory transitions to transform the time-varying polynomial into a constant, which enables the deterministic component predictable. However, these methods [14,15,16,17,18] are still challenging to perform long-term prediction since excessive gate transitions would cause the loss of representations.

In addition to the recurrent models, other models are also employed for spatiotemporal prediction. A retrospection network is proposed in [19], which introduces retrospection loss to push the retrospection frames to be consistent with the observed frames. In order to handle the imbalance in the data, a neighborhood cleaning algorithm is developed in [20]. A random forest algorithm extracts the optimal features to perform prediction task. A variational autoencoder is adopted to extract nonlinear dynamic features in [21]. This model analyzes the correlations between variables and the relationships between historical samples and present samples. A wide-attention module and the deep-composite module are utilized in [22] to extract global key features and local key features. However, these methods [19,20,21,22] depend on local representations to some extent, which cannot get excellent performance on prediction task. An artificial neural network [23] has been proposed to model the unique properties of spatiotemporal data and derives a more powerful modeling capability to spatiotemporal data. A spatiotemporal prediction system [24] has been developed to focus on spatial modeling and reconstructing the complete spatio-temporal signal. This method shows the effectiveness of modelling coherent spatio-temporal fields. Mixpred neural network has been proposed to model the dynamic pattern and learn appearance representations based on given video frames in [25]. A 3D CNN is utilized into RNN in [26], which extends representations in temporal dimension and makes the memory unit store better long-term representations. However, convolutional operations [24,25,26] account for short-range intraframe dependencies due to their limited receptive fields and the lack of explicit inter-frame modeling capabilities. The generative adversarial networks [8] is another approach for spatiotemporal prediction. A conditional variational autoencoder method has been proposed in [9] by producing future human trajectories conditioned on previous observations and future robot actions. The prediction methods [8,9] aim to generate less blurry frames, but their performance significantly depends on the unstable training process.

A self-attention mechanism is proposed in [27], which can be applied to capture long-range dependencies and has been proved to be effective in aggregating salient features among all spatial positions in computer vision tasks [28,29,30]. A double attention block is proposed in [28], which combines the features of the whole space into a compact set, and then adaptively selects and allocates features to each location. In order to exploit the contextual information more effectively, a crisscross network [29] introduced a crisscross attention module to get the contextual information of all pixels, which is helpful for visual understanding problems. In addition, unlike the multi-scale feature fusion methods, a dual attention network [30] is proposed to combine local features with global dependencies adaptively. However, they cannot be used to deal with prediction tasks due to the lack of spatiotemporal dependencies.

In summary, prior prediction models yield different drawbacks. Different from previous work, we design a novel variant of ConvLSTM [5] to store state representations and extend the attention mechanism in the task of spati otemporal prediction. This architecture captures rich contextual relationships for better feature representations with intra-class compactness.

Table 1 shows the acronyms used in the paper with a definition about the concept.

## 3. DMA Neural Network

**Figure 1:**
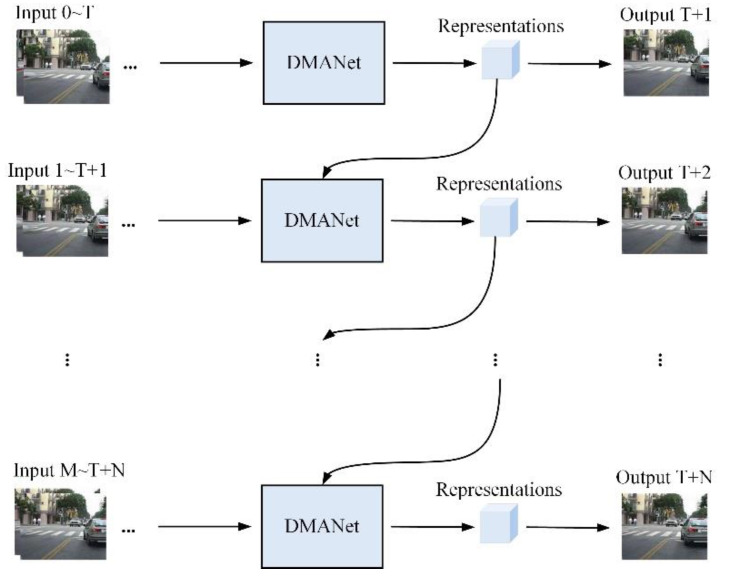
A flow chart of DMANet in long-term prediction.

A flow chart of DMANet is shown in Figure 1. The representations are extracted from DMANet given the input frames. The representations indicate prediction result and can be used to predict the next representations.

In this section, the details of the DMANet would be given. Firstly, a novel DMLSTM unit is introduced in Section 3.1. Afterwards, a dual attention mechanism is proposed in Section 3.2, which enables the model can benefit from the previous relevant representations. Finally, they are aggregated together to build DMANet for spatiotemporal prediction, which is detailed in Section 3.3.

### 3.1. Dual Memory LSTM

It is enlightened by the PredRNN++ [7], which adds more nonlinear layers to increase the network depth and strengthen the modeling capability for spatial correlations and temporal dynamics. However, the problem of gradient propagation is becoming more and more difficult with the increase of network depth, even if GHU [7] alleviates it to a limited extent. Some work [6,7,14] does not perform well in extracting the representations of spatiotemporal sequences across excessive gate transitions, as it may inescapably cause the loss of representations. Therefore, long-range spatial dependencies can be captured by stacked convolution layers. However, the effectiveness of the modeling capability for spatiotemporal dynamics is limited due to the complex layer-to-layer transition.

**Figure 2:**
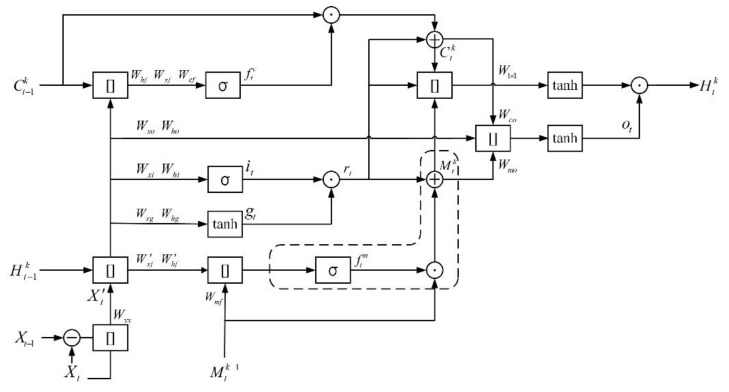
DMLSTM unit.

A new recurrent unit named DMLSTM is developed to perform spatiotemporal prediction to overcome the limitations as mentioned above, as shown in Figure 2. Firstly, an additional memory unit is added based on ConvLSTM [5]; this unit is used to store spatial states, which enables the unit to learn more spatiotemporal representations. The novel transition mechanism is designed by discarding redundant gate structure, such as input gate. The various nonlinear structure would loss the powerful internal representations in pixel-level prediction. On the other hand, the representations differencing operations has been effectively applied to capture the representations of moving objects. Therefore, differencing can be used for prediction task to supplement moving objects representation details. In the DMLSTM unit, the differencing operation is developed to get representations of motion by differencing adjacent hidden states or raw images, which makes the unit have a more powerful modeling capability for spatiotemporal dynamics.

In the developed DMLSTM unit, [] denotes a concatenation operator, *σ* is the sigmoid activation function, tanh is the activation function, ⊙ is the Hadamard product, ⊕ is element-wise addition, and ⊖ is element-wise difference. All vectors are represented in bold. $C_{t}^{k}$ is temporal memory states, and $M_{t}^{k}$ is spatial memory states, where *k* indicates the *k*th hidden layer and *t* denotes time stamp. ***f***_*c t*_, ***f***_*m t*_ are forget gate, respectively, where the superscript *c* and *m* denote the forget gate are used in temporal memory $C_{t}^{k}$ and spatial memory $M_{t}^{k}$, respectively. ***i****_t_* is input gate, ***g****_t_* is input modulation gate, and ***o****_t_* is output one, respectively. ***X***′ is differential features, ***r****_t_* is input result, and $H_{t}^{k}$ denotes the output of DMLSTM unit, respectively. There are five inputs including the input image features ***X****_t_* from encoders, the spatial memory states $M_{t}^{k-1}$ from previously hidden layers, the input image features ***X****_t_*_−1_ conveyed from encoders at the last time step, the temporal memory states $C_{t-1}^{k}$ and the hidden states $H_{t-1}^{k}$ delivered from previous time step, respectively. All of them are three dimensional tensors in ℝ*^H × W × C^*, where *H* and *W* are spatial size and *C* denotes the number of channels, respectively. The update equations of DMLSTM unit are as follows:

(1) $${X^{\prime }}_{t}=W_{xx}*\left[\left(X_{t}-X_{t-1}\right),X_{t}\right]+b_{x}$$

where ∗ is the convolution operation, and [] indicates concatenation of the tensors. ***W***_xx_ is the convolutional filters. $b_{x}$ is the bias vector.

Since the moving objects has strong correlations between consecutive images, the proposed unit is applied to learn the inner dynamics of the movement by taking differencing operations between two consecutive images features. The representations of moving objects concatenated with frame features ***X****_t_* to enrich input representations according to (1).

(2) $$f_{t}^{c}=\sigma \left(W_{xf}*X_{t}^{\prime }+W_{hf}*H_{t-1}^{k}+W_{cf}*C_{t-1}^{k}+b_{f}\right)$$

(3) $$f_{t}^{m}=\sigma \left(W_{xf}^{\prime }*X_{t}^{\prime }+W_{hf}^{\prime }*H_{t-1}^{k}+W_{mf}*M_{t}^{k-1}+b_{f}^{\prime }\right)$$

(4) $$i_{t}=\sigma \left(W_{xi}*X_{t}^{\prime }+W_{hi}*H_{t-1}^{k}+b_{i}\right)$$

(5) $$g_{t}=tanh\left(W_{xg}*X_{t}^{\prime }+W_{hg}*H_{t-1}^{k}+b_{g}\right)$$

where $W_{xf}$, $W_{hf}$, $W_{cf}$, *, **W***_*mf*_, $W_{xi}$, $W_{hi}$, $W_{xg}$, $W_{hg}$, $W_{xf}^{\prime }$, and $W_{hf}^{\prime }$ are convolutional filters, respectively. ***b****_f_*, ***b****_i_*, ***b****_g_*_,_ and $b_{f}^{\prime }$ are the bias vectors, respectively.

Some previous work [6,7] tended to extract the representations across excessive gate transitions, which would cause the loss of representations. An extra forget gate ***f***_*m t*_ and spatial memory states $M_{t}^{k}$ are added based on standard ConvLSTM [5], as shown in Figure 2 (dotted part). The forget gate ***f***_*m t*_ is used to forget representations that are not relevant to $M_{t}^{k}$. The spatial memory states $M_{t}^{k}$ is used to store spatial representations for further use. The forget gate ***f***_*c t*_ and ***f***_*m t*_, the input gate ***i****_t_* and the input modulation gate ***g**_t_* are controlled through hidden states $H_{t-1}^{k}$, the differential features ***X***′, previous memory states $C_{t-1}^{k}$ and $M_{t}^{k-1}$ are gotten according to (2) to (5). Such a transition mechanism extracts the representations by simpler gate structures to avoid representations loss in massive gates transition.

(6) $$r_{t}=i_{t}\cdot g_{t}$$

(7) $$C_{t}^{k}=f_{t}^{c}\cdot C_{t-1}^{k}+r_{t}$$

(8) $$M_{t}^{k}=f_{t}^{m}\cdot M_{t}^{k-1}+r_{t}$$

where ∗is the Hadamard product. These memory states $C_{t}^{k}$, $M_{t}^{k}$ depends on previous memory states and input result ***r****_t_* according to (6) to (8), which could make unit obtain current states based on the current learning input and previous states.

(9) $$o_{t}=tanh\left(W_{xo}*X_{t}^{\prime }+W_{ho}*H_{t-1}^{k}+W_{co}*C_{t}^{k}+W_{mo}*M_{t}^{k}+b_{o}\right)$$

(10) $$H_{t}^{k}=o_{t}\cdot tanh\left(W_{1\times 1}*\left[r_{t},C_{t}^{k},M_{t}^{k}\right]\right)$$

where $W_{xo}$, $W_{ho}$, $W_{co}$*,* and $W_{mo}$ are convolutional filters, respectively. $W_{\times 1}^{1}$ is a 1 × 1 convolutional filter for dimension reduction. ***b****_f_* and ***b****_o_* are the bias vectors, respectively. These memory states $C_{t}^{k}$ and $M_{t}^{k}$ are concatenated with input result ***r****_t_* to get the future hidden states $H_{t}^{k}$ through output gate ***o****_t_* according to (9) and (10).

Given an input frame, the goal of our unit is to predict diverse plausible future frames as mentioned above. The unit firstly performs a process of differencing operations between the neighboring frames to produce differential representations containing movement information of objects. The differential representations are concatenated with input to enrich spatiotemporal information for further use. In the next stage, the concatenated representations are fed to the unit to predict future representations. The DMLSTM unit includes both temporal and spatial memories to storage spatiotemporal representations for future prediction. The unit can be applied to generate a candidate of the next frame based on extracted spatiotemporal representations.

### 3.2. Dual Attention Mechanism

Spatiotemporal prediction can predict future frames by observing previous representations. However, the prediction model should focus more on historical representations that is related to the predicted content. Attention mechanism [27] can capture long-range dependences between local and global representations in some practical tasks [32,33]. Moreover, spatiotemporal prediction is challenging due to the complex dynamics and appearance changes, which requires dependencies on both temporal and spatial domains. A novel variant of attention mechanism named dual attention mechanism is proposed. This architecture captures long-term spatiotemporal interaction from temporal and spatial dimensions, respectively, and then the obtained representations are aggregated for future prediction.

**Figure 3:**
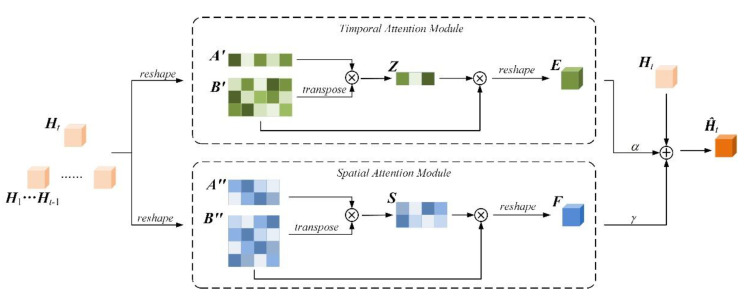
Dual attention module.

The dual attention module is shown in Figure 3 including current time stamp hidden states ***H****_t_* ∈ ℝ*^H × W × C^* and historical ones {***H***_1_…***H****_t_*_−1_} ∈ ℝ*^n × H × W × C^*, where *H* and *W* are spatial size, *C* is the number of channels, and *n* denotes the number of hidden representations that are concatenated along the temporal dimension, respectively.

For the temporal attention module, ***H****_t_* and {***H***_1_…***H****_t_*_−1_} are reshaped into ***A***′ ∈ ℝ^1 *× HWC*^ and ***B***′ ∈ ℝ*^n × HWC^*, respectively. A matrix multiplication is performed between ***A***′ and the transpose of ***B***′. A softmax function is applied to get the temporal attention map ***Z*** ∈ ℝ^1 *× n*^:

(11) $$z_{1j}=\frac{exp\left({A^{\prime }}_{1}⊗{\left(B_{j}^{\prime }\right)}^{T}\right)}{\sum_{j=1}^{n}exp\left(A_{\prime }^{1}⊗{\left(B_{j}^{\prime }\right)}^{T}\right)}$$

where ⊗ is matrix multiplication operator, the superscript *T* indicates matrix transpose, $A_{1}^{\prime }$ ∈ ℝ^1 *× HWC*^ and $A_{\prime }^{1}$ = ***A***′ due to only one hidden states representation, $B_{j}$ ∈ ℝ^1 *× HWC*^, and ***z***_1*j*_ indicates the temporal similarity score between the current time stamp representations and the previous *j*th time stamp representations. The more relevant representations of the two time stamps contribute to greater the weights on attention map.

A matrix multiplication is performed between ***Z*** and ***B***′ to get the temporal attention module output ***E***∈ ℝ^1 *× H × W × C*^ as follows:

(12) $$E=\sum_{j=1}^{n}z_{1j}⊗B_{j}^{\prime }$$

Similarly, in the spatial attention module the representations ***A***′′ ∈ ℝ*^HW × C^*, ***B***′′ ∈ ℝ*^nHW × C^* is reshaped from original representations ***H****_t_* and {***H***_1_…***H****_t_*_−1_}, a matrix multiplication and softmax function is applied to get the spatial attention map ***S*** ∈ ℝ*^HW × nHW^*:

(13) $$s_{ji}=\frac{exp\left(A_{i}^{\prime \prime }⊗{\left(B_{j}^{\prime \prime }\right)}^{T}\right)}{\sum_{j=1}^{n}exp\left(A_{i}^{\prime \prime }⊗{\left(B_{j}^{\prime \prime }\right)}^{T}\right)}$$

where **$A_{i}^{\prime \prime }$** ∈ ℝ^1 *× C*^ and **$B_{j}^{\prime \prime }$** ∈ ℝ^1 *× C*^. ***s****_ji_* indicates the spatial similarity between *i*th position at the current time stamps representations and the *j*th position at the historical records ones.

A matrix multiplication is employed between ***S*** and ***B***′′ to get the spatial attention module output ***F*** ∈ ℝ^1 *× H × W × C*^ as follows:

(14) $$F=\sum_{j=1}^{n}s_{ij}⊗B_{j}^{\prime \prime }$$

In pursuit of utilizing the contextual information generated by these two attention modules and ensuring the dual attention module is stable to be embedded into DMLSTM unit, these representations are aggregated, and residual mechanism is applied. The aggregated representation ***Ĥ****_t_* ∈ ℝ^1 *× H × W × C*^ is calculated as follows:

(15) $${\hat{H}}_{t}=\alpha E+\gamma F+H_{t}$$

where *α* and *γ* is used to weight the contribution of ***E*** and ***F***, respectively. Both *α* and *γ* would be discussed later.

**Figure 4:**
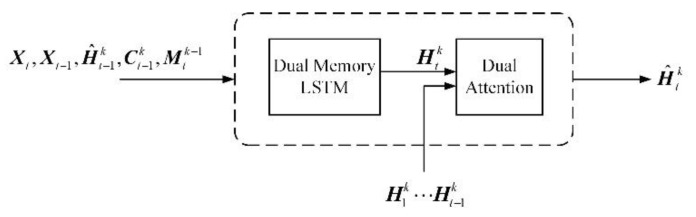
DMA unit.

The dual attention memory module is embedded into the DMLSTM unit to construct the DMA unit, as illustrated in Figure 4. The operations in DM-LSTM are followed by Equations (1)–(10). The DMLSTM unit can be applied to characterize the features of input frames, which is discussed later. The operations in Dual Attention are followed by Equations (11)–(15). The dual attention module can adaptively memorize the longer dependences by aggregating long-term contextual information.

### 3.3. DMANet

**Figure 5:**
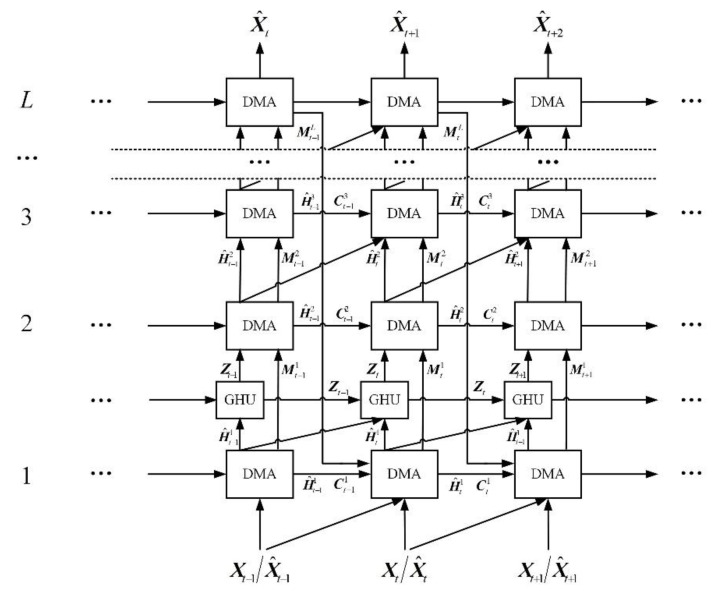
DMANet.

In order to design a powerful spatiotemporal prediction model, a DMANet is built by stacking *L* DMA units to extract highly abstract representations. In addition, the GHU [7] is injected between the 1st and 2nd layers to alleviate the problem of vanishing gradient. The prediction result is generated by mapping the output representations back to the pixel value space. A schematic of the developed DMANet is shown in Figure 5. The calculations of the entire model are as follows (for 3 ≤ *k* ≤ *L*):

(16) $${\hat{H}}_{t}^{1},M_{t}^{1},C_{t}^{1}=\mathrm{DMA}\left(X_{t},X_{t-1},{\hat{H}}_{t-1}^{1},C_{t-1}^{1},M_{t}^{L}\right)$$

(17) $$Z_{t}=\mathrm{GHU}(Z_{t-1},{\hat{H}}_{t}^{1})$$

(18) $${\hat{H}}_{t}^{2},M_{t}^{2},C_{t}^{2}=\mathrm{DMA}\left(Z_{t},{\hat{H}}_{t-1}^{1},{\hat{H}}_{t-1}^{2},C_{t-1}^{2},M_{t}^{1}\right)$$

(19) $${\hat{H}}_{t}^{k},M_{t}^{k},C_{t}^{k}=\mathrm{DMA}\left({\hat{H}}_{t}^{k-1},{\hat{H}}_{t-1}^{k-1},{\hat{H}}_{t-1}^{k},C_{t-1}^{k},M_{t}^{k-1}\right)$$

where the superscript *L* denotes the number of DMANet layers, which would be discussed later. The subscript *t* denotes the time stamp. ***Z****_t_* denotes hidden states from GHU [7], which models long-term dynamics according to (17).

The input frames ***X***_t_ are fed into the bottom layer to predict future ones. The hidden representations ***Ĥ****_t_* horizontally and vertically transmitted. The diagonal arrows denote the forward directions of ***X***_*t*_ or ***Ĥ****_t_* for differential modeling. The memory states $C_{t-1}^{k}$ horizontally conveyed from *t*−1 stamp to *t* one. $C_{t-1}^{k}$ is used to store temporal representations at *t*−1 stamp. The memory states $M_{t}^{k-1}$ vertically delivered from *k*−1 layer to *k* one. $M_{t}^{k-1}$ is used to store spatial representations at *k*−1 layer. Specially, the memory states $M_{t}^{L}$ would be updated in a zigzag direction at top layer, as $M_{t}^{1}$= $M_{t-1}^{L}$ in which the DMA units can be applied to get more sufficient representations of past for further prediction. The final output ${\hat{X}}_{t+1}$ indicates prediction result and can be used to predict next representations.

Since this structure utilizes several state transitions paths to deliver the extracted representations which is necessary for spatiotemporal prediction, the stacked DMANet could be applied to extract more high-level representations from the bottom layer upwards. Besides diagonal state transition paths are exploited to extract motion representations of moving objects by differencing operations. The developed DMANet can be applied to get both spatiotemporal representations and capture the longer dependences by DMLSTM unit and dual attention module, respectively.

### 3.4. Training Method

*L*1 and *L*2 losses has been widely used for prediction task [6,7]. *L*1 loss can alleviate blurry prediction results. *L*2 loss can make the model converge faster. For training, the loss function used is the sum of L1 and L2 terms to optimize DMANet and they are combined as follow:

(20) $$L\left(\hat{Y},Y\right)=\sum_{i=1}^{n}\left(\left|{\hat{Y}}_{i}-Y_{i}\right|+\frac{1}{2}{\left|{\hat{Y}}_{i}-Y_{i}\right|}^{2}\right)$$

where |·| is absolute value function operator; *n* is the number of prediction frames. ***Ŷ*** and ***Y*** denote the prediction results and the ground truth, respectively. *Ŷ_i_* and *Y_i_* are the *i*th element of ***Ŷ*** and ***Y***, respectively.

## 4. Experiments

### 4.1. Dataset and Implements

All experiments are implemented using TensorFlow on a Linux machine equipped with an Intel Xeon E5-2683 v3 CPU and Nvidia GeForce GTX 1070Ti GPU. In order to verify the performance of the proposed method, the experiments are performed on some challenging datasets. To test the performance of the developed method, some datasets are selected as follows. Moving MNIST [34] is constructed by two digits moving independently around the frame. The digits are placed initially at random locations. The movement of digits is irregularly, which makes model difficult to maintain the accuracy of predictions. Moving MNIST [34] contains 10,000 sequences for training set and 5000 sequences for test set. Each sequence consists of 20 frames with 10 for inputs and 10 for prediction results, and each frames size are 64 × 64 × 1.

KITTI [35] is another tested dataset, which is taken by the vehicle-mounted camera on a car driving around an urban environment. The “City”, “Residential”, and “Road” categories are selected for training. To further assess the performance of the developed method with robust representation, the trained model is tested on the Caltech [36], which is another car-mounted camera video dataset. These datasets describe rich temporal dynamics of multiple moving objects and presents another level of difficulty for spatiotemporal prediction. The model evaluated on the Caltech [36] by predicting 10 future frames given 10 previous frames. The training set consisted of 40,312 sequences. The tested set contains 3631 sequences. All sequences include 20 frames, which are center cropped and downsampled to 128 × 160 × 3.

**Figure 6:**
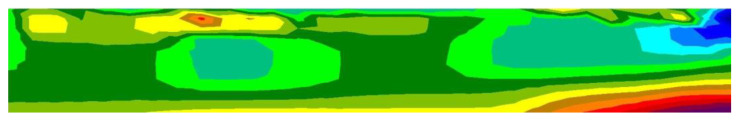
An example of apparent resistivity maps from Yungang Grottoes.

Another dataset called as apparent resistivity (AR) one is selected to test the performance of the developed method. AR dataset is obtained from Chinese Yungang Grottoes, which is a world-famous treasure house of Buddhist art. It is completely different from the previous datasets. Since grotto cultural relics are vulnerable to water, we have carried out the work of high-density electric prospecting for the water source in the grottoes to protect effectively the cultural relics. We designed a cable with 32 electrodes above the grottoes. In order to reduce the contact resistance, the electrode was coated with soaked bentonite. Each electrode is separated by 2 m and buried in a 20 cm pit. There are various electrode arrays constructed by 4 electrodes, which are used to measure resistivity data at different depths. The cable is connected with the ABEM instrument to get resistivity data. The resistivity data contained 155 wenner arrays and 223 gradient arrays. The resistivity data is inversed by Res2Dinv soft to get apparent resistivity map as shown in Figure 6. The different colors represent different intensities of resistivity. The redder the color, the higher of resistivity, which indicates there is less likely to contain water. The bluer the color, the opposite. One can find from Figure 6 that the apparent resistivity map includes various resistivity sections, which means there would be several trends of resistivity. The intensity of resistivity is affected easily by the weather, which could cause vagaries in apparent resistivity maps. These properties make the prediction of resistivity change is difficult.

The apparent resistivity data are recorded every 8 h based on the regular pattern of resistivity change. We carried out continuous field high-density electrical monitoring for about one month. To enhance short time resistivity variations and network samples, we adopted B-spline interpolation [37] for the measured apparent resistivity data. The B-spline interpolation is as follows:

(21) $$C\left(t\right)=\sum_{i=0}^{n-1}B_{i,p}\left(t\right)P_{i}$$

(22) $$B_{i,p}\left(t\right)=\frac{t-t_{i}}{t_{i+p}-t_{i}}B_{i,p-1}\left(t\right)+\frac{t_{i+p+1}-t}{t_{i+p+1}-t_{i+1}}B_{i+1,p-1}\left(t\right)$$

(23) $$B_{i,0}=\{\begin{array}{c} 1,\\ 0, \end{array}\begin{array}{c} if\;t_{i}\leq t\leq t_{i+1}\\ otherwise \end{array}$$

where *t* is timestamp. *n* is the number of control point; *n* in (21) is set as 74 because there are 74 timestamps. *P_i_* is *i*th control point. *p* is interpolation order, which is set to 2 to eliminate linear noise and the effects of baseline drift. *B_i_*_,*p*_(*t*) is parameters of basic function. *C*(*t*) represents the interpolation result with time.

**Figure 7:**
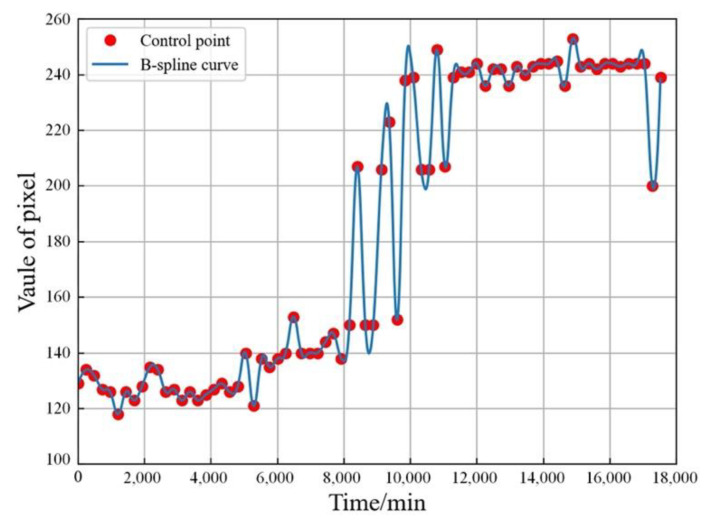
The example of B-spline curve.

The produced B-spline curve as shown in Figure 7. The B-spline interpolation [37] is used to enhance apparent resistivity map data to be a continuous derivative curve for better matching the data requirement. One can find that the curve perfectly fits the change of control point, which indicates that the cumulative change of resistivity can be represented by the B-spline interpolation [37]. Each frame is captured at an interval of 20 min. Our apparent resistivity maps contains 17,520 frames. According to the disjoint principle, the dataset for the apparent resistivity is divided into training set and test set with 15,748 and 1732 sequences, respectively. Each sequence contains 10 frames for input and 10 frames for prediction results.

In our experiments, the learning rate and batch size are set to 0.001 and 8, respectively. The model is trained 100,000 iterations. All models predict next frames from previous 10 observations. Then, sliding window of one step stride is adopted to predict future 10 frames. For the evaluation metrics, the mean square error (MSE), peak signal to noise ratio (PSNR) and structural similarity index measure (SSIM) [31] are used to measure the quality of reconstruction. MSE is used to evaluate the difference between the prediction result and the ground truths. PSNR is adopted to evaluate the predicted image quality. SSIM is used to evaluate the similarity between the prediction result and the ground truths. All metrics are averaged over the predicted frames. The lower MSE or the higher SSIM denotes the smaller difference between the prediction results and the ground truth. PSNR emphasizes the foreground appearance, the higher PSNR indicates the better quality of prediction results.

### 4.2. Parameter Analyses

**Figure 8:**
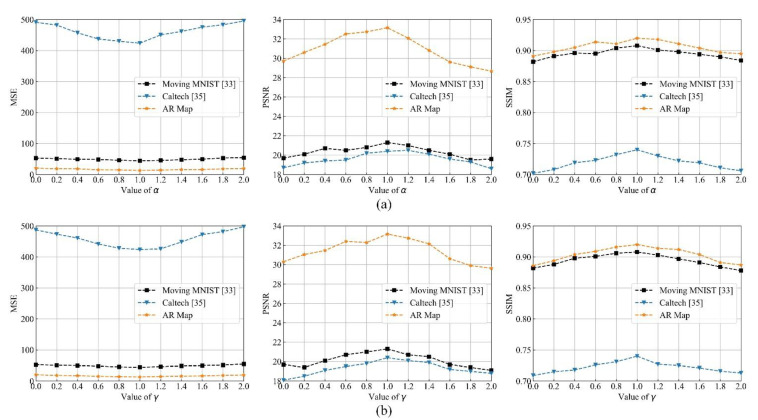
Prediction performance in different αs (**a**) and γs (**b**) at different datasets from top to bottom, left to right, respectively.

The contextual information generated by temporal attention module and spatial attention module are aggregated as (15). To get a reasonable value of *α* and *γ* in (15), the value of *α* is changed from 0 to 2 at an interval of 0.2. The value of *γ* was set 1. Meanwhile, the number of hidden layers was set 4 with the channel 128, 64, 64, 64. The developed method is evaluated on the datasets as mentioned above. The results are shown in Figure 8a. One can find that *α* is set 1 to get trade-off between MES and MAE and kept the same in the subsequent experiments.

Similarly, the value of *γ* is changed from 0 to 2 at an interval of 0.2. The value of *α* was set 1. The number of hidden layers and channel as mentioned above. The experimental results are shown in Figure 8b. When *γ* is 1, the prediction performance is the best. In the subsequent experiments, *γ* is set as 1 and keep the same.

**Figure 9:**
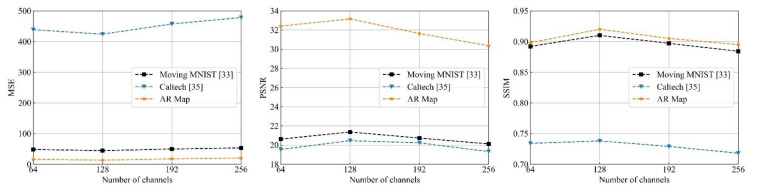
Prediction performance under different number of channels in bottom layer.

The number of hidden channels is another factor in representations extraction for spatiotemporal prediction. Low-level representations have a strong impact on the prediction result of DMANet. The representations may not be extracted at all, or the network performance is poor if the number of hidden channels is too small. However, if the number of channels in the hidden layer is too great, the error would be increased, and the training time of the whole network model would be prolonged. In order to get an optimal number of hidden channels in bottom layer, the number of hidden channels is changed from 64 to 256 at an interval of 64. Then, the number of hidden layers is fixed to 4 and the number of channels in all layers except the bottom layer is set to 64. The comparison results are shown in Figure 9. It can be seen from Figure 9 that the number of channels in the bottom layer has significant influence on the prediction performance. When the number of channels in bottom layer is 128, the prediction performance is the best. Then, prediction performance decreases with channel increasing. Therefore, the number of hidden channels in bottom layer is set as 128 and kept the same in the subsequent experiments.

**Figure 10:**
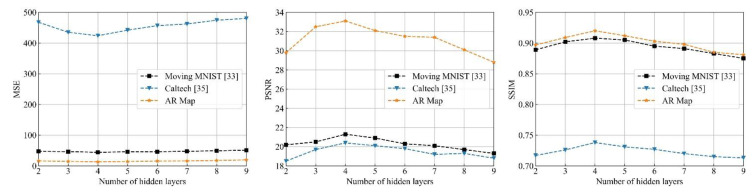
Prediction performance under different number of hidden layers.

On the other hand, DMANet is constructed by stack *L* DMA units. Deeper networks can capture spatiotemporal representations more effectively. To get a reasonable number of hidden layers for DMANet, the number of hidden layers is changed from 2 to 9 at an interval of 1. The proposed model is evaluated on the datasets as mentioned above with different number of layers. The comparison results are shown in Figure 10. One can find that with the increase of the number of hidden layers, the prediction performance increases gradually at first. When the number of hidden layers is 4, the prediction performance is the best. Then, the prediction performance decreases gradually with the increase of hidden layers. The reason is that the prediction model can be further extract video representations with the increase of the number of hidden layers, but excessively layers may inevitably lead to training difficulty and a loss of information representations. In the subsequent experiments, the number of DMANet layers is set 4 and kept same in the subsequent experiments.

### 4.3. DMLSTM Unit and the Dual Attention Mechanism Evaluation

To assess the effectiveness of both DMLSTM unit and the dual attention mechanism, four variants of our model are applied including: PredRNN++ [7] is taken as a baseline model. DMLSTM is consisted of stacking 4-layer DMLSTM units. DA-PredRNN++ is PredRNN++ [7] with the dual attention. DMANet is built by stacking 4-layer DMA units. Some results are given in Table 2.

One can find from Table 2 that the developed DMANet achieves the best result on all datasets by comparisons. The reason is that DMANet adopts a new transition mechanism and differencing operations, which could more effectively extract the representations of spatiotemporal sequences and the motion trend of objects. In addition, DMANet is optimal as the dual attention mechanism could make full use of the spatiotemporal contextual dependences. The attention mechanism is utilized to obtain global representations, which is a practical way to improve prediction performance. The experiment results demonstrate that the proposed DMLSTM unit and dual attention mechanism has excellent prediction performance.

### 4.4. Comparisons with Some State-of-the-Art Methods

In order to further evaluate whether the proposed method is effective to perform prediction, the proposed method has been compared with some methods [6,7,14,18]. PredRNN [6] and PredRNN++ [7] introduced a zigzag memory flow and GHU to alleviating gradient vanishing. FRNN [14] is an architecture based on recurrent convolutional autoencoders, which can address the network capacity and error propagation problems for future prediction. MIM [18] captures higher orders of non-stationarity to facilitate non-stationarity modeling and make the future sequence more predictable. The parameter used are all those recommended by the authors in [6,7,14,18], respectively. Some comparison results as follows.

**Figure 11:**
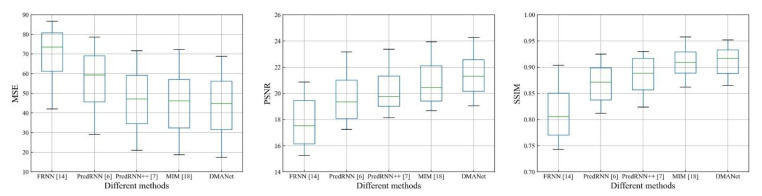
Whisker plot comparisons of the different models at the Moving MNIST [34].

**Figure 12:**
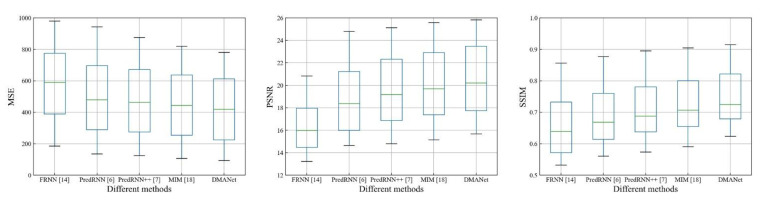
Whisker plot comparisons of the different models at the Caltech [36].

**Figure 13:**
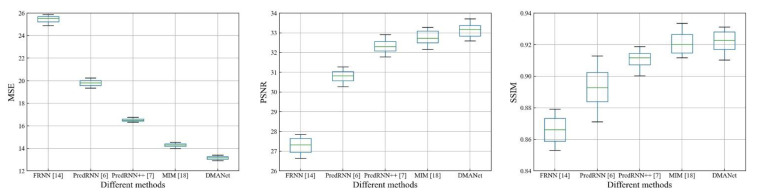
Whisker plot comparisons of the different models at the AR Map.

Figure 11, Figure 12 and Figure 13 shows whisker plot comparisons at the chose datasets, which are used to reflect the distribution characteristics of the prediction results. It can be seen from Figure 11, Figure 12 and Figure 13 that the developed method achieves the best performance with statistical significance among the investigated methods.

**Figure 14:**
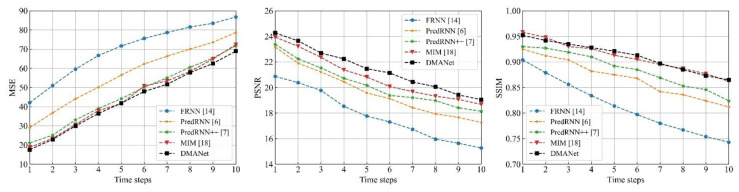
Frame-by-frame quantitative results for the 10 frames at the Moving MNIST [34].

**Figure 15:**
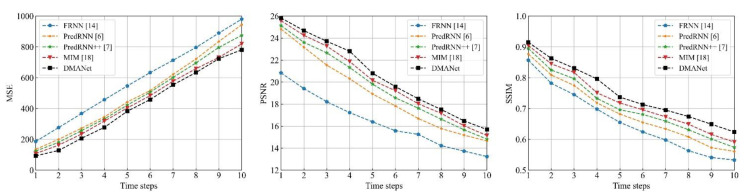
Frame-by-frame quantitative results for the 10 frames at the Caltech [36].

**Figure 16:**
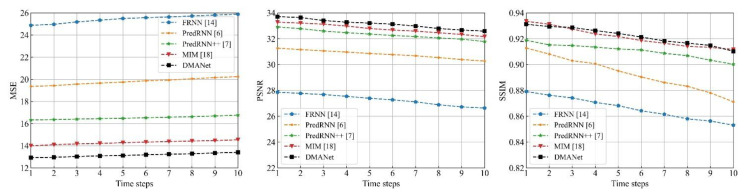
Frame-by-frame quantitative results for the 10 frames at the AR Map.

Figure 14, Figure 15 and Figure 16 shows frame-by-frame quantitative experiments for the 10 frames at the chose datasets. It can be seen that the developed method has the best performance among the investigated methods with the lowest MSE, both the highest PSNR and SSIM at each frame.

To further demonstrate that the proposed method has the best performance, we have computed results as mean ± standard deviation in Table 3. One can find from Table 3 that the proposed method has the best performance among the investigated methods. Some reasons are as follows. A bijective mapping method is utilized to share states between encoder and decoder in [14], bijective mapping could extract representations from low dimension to high dimension. However, the relationship between the consecutive representations is not considered, which is important to dynamic objects modeling. PredRNN [6] is not able to forecast accurately due to vanishing gradient and inefficient representations. The dynamic regions are blurred, and the action of objects is uncertain due to inefficient representations. The problem of vanishing gradient indicates that PredRNN [6] cannot maintain accuracy and image quality when carrying out long-term prediction. PredRNN++ [7] increases the transition depth to improve prediction performance. However, it would cause a loss of representations during recurrent memory transitions. Inefficient representations cause the blurring effect of PredRNN++ [7]. MIM [18] utilizes differencing operations to reduce the order of non-stationary polynomials and focuses more on the non-stationary dynamics, which is effective for spatiotemporal prediction. However, it is not able to explicitly distinguish multiple objects in some particular scene. The proposed method could effectively extract the representations of spatiotemporal sequences and capture moving objects by DMLSTM unit to solve these drawbacks. On the other hand, the long-term spatiotemporal dependences are extracted by dual attention mechanism. There are sufficient representations utilized to get better prediction results. The experimental results show that the proposed method has excellent performance for spatiotemporal prediction.

## 5. Conclusions

A DMANet has been proposed for spatiotemporal prediction in this paper. A DMLSTM unit is used to efficiently extracts the representations by leveraging differencing operations between the consecutive images and adopting a dual memory transition mechanism. A dual attention mechanism is designed to captures long-term spatiotemporal dependences by compute the correlations between the current hidden representations and the historical hidden representations from temporal and spatial dimensions, respectively. The DMANet combines both the advantages, and such architectural design enables the model with greater modeling power for short-term dynamics and long-term contextual representations. The experimental results demonstrate that our method has excellent performance in spatiotemporal prediction.

Spatiotemporal prediction is a promising avenue for the self-supervised learning of rich spatiotemporal correlations. For future work, we will investigate how to separate the moving objects from the background and put more attention on moving objects. We will also try to build an apparent resistivity nowcasting system to protect Chinese Grottoes from water.

## Figures and Tables

**Table 1:** The acronyms with a definition about the concept.

Acronym	Describe
DMANet	DMANet is Dual Memory LSTM with Dual Attention Neural Network.
DMLSTM	DMLSTM is Dual Memory LSTM.
ConvLSTM [5]	ConvLSTM is Convolutional LSTM.
GHU [7]	GHU is Gradient Highway Unit.
RNN [10]	RNN is Recurrent Neural Network.
LSTM [11]	LSTM is Long Short-Term Memory.
MIM [18]	MIM is Memory in Memory Network.
AR	AR is Apparent Resistivity.
MSE	MSE is Mean Square Error.
PSNR	PSNR is Peak Signal to Noise Ratio.
SSIM [31]	SSIM is Structural Similarity Index Measure.

**Table 2:** Ablation study in different methods.

Dataset	Moving MNIST [34]			Caltech [36]			AR Map		
	MSE	PSNR	SSIM	MSE	PSNR	SSIM	MSE	PSNR	SSIM
PredRNN++ [7]	46.51	20.22	0.88	479.26	19.57	0.71	16.54	32.35	0.91
DMLSTM	49.66	20.53	0.90	437.65	20.14	0.72	18.45	32.78	0.90
DA-PredRNN++	46.23	20.86	0.90	442.22	19.75	0.73	15.17	32.56	0.91
DMANet	44.36	21.36	0.91	423.98	20.46	0.74	13.14	33.16	0.92

**Table 3:** Comparisons with different methods.

Dataset	Moving MNIST [34]			Caltech [36]			AR Map		
	MSE	PSNR	SSIM	MSE	PSNR	SSIM	MSE	PSNR	SSIM
FRNN [14]	69.76 ± 14.01	17.83 ± 1.91	0.81 ± 0.05	587.83 ± 251.22	16.43 ± 2.37	0.66 ± 0.11	25.48 ± 0.32	27.23 ± 0.41	0.86 ± 0.01
PredRNN [6]	58.82 ± 15.58	19.66 ± 1.86	0.86 ± 0.04	503.84 ± 259.64	18.83 ± 3.31	0.69 ± 0.10	19.81 ± 0.28	30.81 ± 0.31	0.89 ± 0.02
PredRNN++ [7]	46.51 ± 16.18	20.22 ± 1.64	0.88 ± 0.03	479.26 ± 245.43	19.57 ± 3.33	0.71 ± 0.10	16.54 ± 0.13	32.35 ± 0.34	0.91 ± 0.01
MIM [18]	45.24 ± 16.85	20.81 ± 1.72	0.91 ± 0.03	448.51 ± 232.67	20.12 ± 3.64	0.72 ± 0.09	14.27 ± 0.16	32.72 ± 0.37	0.92 ± 0.01
**DMANet**	44.36 ± 16.22	21.36 ± 1.67	0.91 ± 0.02	423.98 ± 233.71	20.46 ± 3.38	0.74 ± 0.09	13.14 ± 0.15	33.16 ± 0.36	0.92 ± 0.01

## Data Availability

Not applicable.
